# Robust Angio-Vasculogenic Properties of 3D-Cultured Dual GCP-2/PDGF-β Gene-Edited Human ASCs

**DOI:** 10.3390/ijms26178425

**Published:** 2025-08-29

**Authors:** Seongho Han, Sang Joon An, Sung-Whan Kim

**Affiliations:** 1 Department of Family Medicine, College of Medicine, Dong-A University, Busan 49236, Republic of Korea; drhans@dau.ac.kr; 2 Department of Neurology, College of Medicine, Catholic Kwandong University, International St. Mary’s Hospital, Incheon 22711, Republic of Korea; neuroan@gmail.com; 3 Department of Medicine, College of Medicine, Catholic Kwandong University, Gangneung 210-701, Republic of Korea

**Keywords:** adipose-derived mesenchymal stem cells (ASCs), angiogenesis, GCP-2, PDGF-β, 3D culture, gene editing

## Abstract

Adipose-derived mesenchymal stem cells (ASCs) have great potential in regenerative medicine due to their abundance and innate multi-lineage differentiation potential. However, the therapeutic efficacy of ASCs is often compromised by poor microenvironmental conditions in the damaged tissues after transplantation. In this study, we generated and assessed genetically modified ASCs that expressed granulocyte chemotactic protein-2 (GCP-2) and platelet-derived growth factor-β (PDGF-β). The results revealed that three-dimensional (3D)-cultured ASCs overexpressing GCP-2 and PDGF-β (3D-A/GP) yielded a significant increase in proangiogenic gene expression, cell migration, and endothelial tube formation in vitro. Moreover, the Matrigel plug assay revealed that 3D-A/GP formed functional blood vessels, and 3D-A/GP injection in a hind limb ischemia (HLI) model revealed higher blood flow recovery, limb salvage, and capillary density and lower apoptosis in mice, compared to the controls. Notably, 3D-A/GP exhibited differentiation into endothelial-like cells and upregulated expression of angiogenic factors in ischemic limb tissue. Our results highlight the value of using a combination of genetic engineering and 3D culture systems to improve the therapeutic effect of ASCs in terms of angiogenesis-dependent tissue repair. The dual modulation of GCP-2 and PDGF-β, in combination with 3D culture, presents a new and synergistic opportunity to maximize the use of ASC-based therapies for ischemic diseases and other regenerative medicine applications.

## 1. Introduction

The use of cell therapy with stem cells from adipose tissue, adipose-derived mesenchymal stem cells (ASCs), in regenerative medicine is a new, innovative approach to treating a variety of diseases that require damaged tissue repair. ASCs are relevant in the field of regenerative medicine due to their availability, abundance, and multi-lineage capacity for differentiation [1]. The therapeutic efficacy of ASCs is due in large part to their paracrine effects, mainly via secretion of angiogenic factors [2]. However, the therapeutic efficacy and survival of ASCs across transplantation procedures are frequently impaired by the microenvironmental conditions in the target tissue [3,4]. Notably, there are indications from recent studies that genetics-modifying approaches to stem cells can increase the therapeutic efficacy of stem cells, including their angiogenic ability [5].

GCP-2 (granulocyte chemotactic protein-2) and PDGF-β (platelet-derived growth factor-β) are two important mediators linked to angiogenesis and tissue repair. GCP-2 is in the CXC chemokine family and has provided much insight into its induction of endothelial cell migration and tubulogenesis via specific molecular pathways [6]. Because GCP-2 has been identified as a key factor in initiating new blood vessel formation and modulating endothelial cell functions, our recent findings suggest that GCP-2 is a strong candidate for enhancing the therapeutic angiogenic potential of ASCs [5]. PDGF-β is also a known factor in vessel maturation and stabilization due to its capability to recruit pericytes to new vessels [7]. Successfully recruiting pericytes and establishing their correct relationships at the right time is a critical aspect to vascular integrity and stabilizing and maintaining new vasculature in the long term. In addition, our recent study demonstrated that the dual integration of angiogenic genes in ASCs enhanced their therapeutic potential [8]. Thus, we hypothesized that GCP-2 is primarily responsible for establishing vessel formation, and PDGF-β could assist in stabilizing newly formed vessels, and all of these factors collaborate to enhance the angiogenic function of ASCs.

Three-dimensional (3D) culture systems are attracting significant attention because they more closely approximate the in vivo microenvironment compared to two-dimensional cultures. Studies have found that stem cells’ survival rate, proliferation, and therapeutic potential can be improved under 3D culture conditions [9,10]. Furthermore, our recent research demonstrated that ASCs cultured in 3D conditions exhibit higher paracrine properties compared to those cultured in 2D conditions. [11]. Thus, combining genetic modification with 3D culture could present a novel strategy for maximizing the angiogenic potential of ASCs in therapeutic applications. In this study, we explored the enhanced angiogenic capacity of ASCs through dual genetic modification involving GCP-2 and PDGF-β within a 3D culture system.

## 2. Results

### 2.1. Targeted Knock-In of GCP-2/PDGF-β in ASCs and Angiogenic Potential of 3D-A/GP

**Figure 1:**
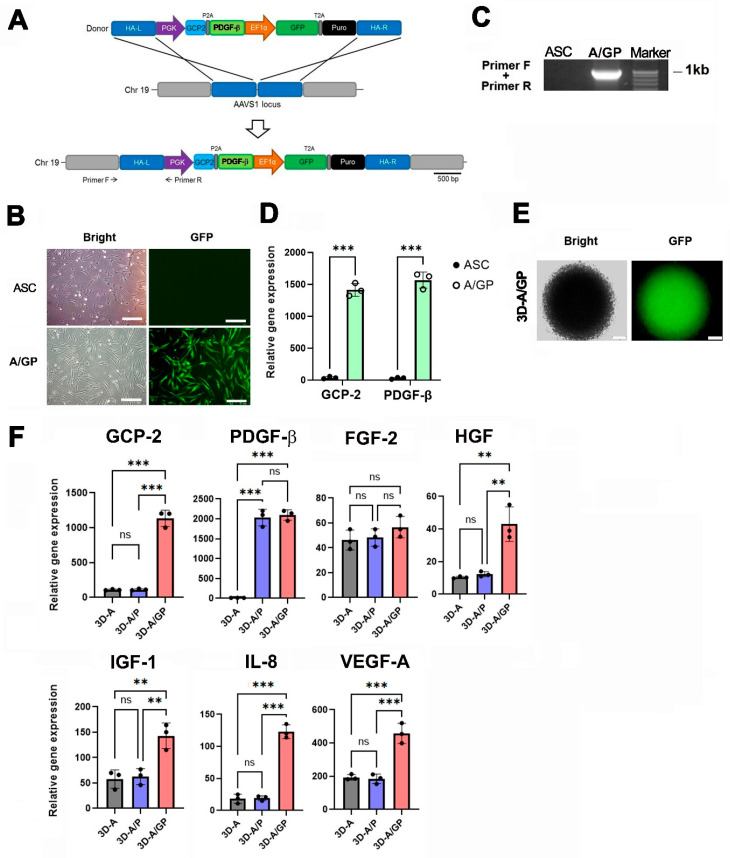
Generation of A/GP cell line and 3D-A/GP using gene editing. (**A**) Schematic of the donor vector carrying the GCP-2 and PDGF-βplasmid DNA. The expression cassette, which includes PGK promoter-driven GCP-2, PDGF-β,and EF1α promoter-driven GFP-T2A-puromycin, was integrated into the AAVS1 locus. Primer locations for junction detection are marked (primers F and R). (**B**) GFP-expressing GP cells. Transfected cells were selected with puromycin and subsequently sorted by FACS. Scale bar = 200 μm. (**C**) Donor plasmid insertion was confirmed via junction PCR. (**D**) qRT-PCR results. GCP-2 and PDGF-βexpression in A/GP compared with untreated ASC control. *** *p* < 0.001; *n* = 3 per group. (**E**) Morphology of GFP-expressing 3D-A/GP. Scale bar = 500 μm. (**F**) Multiple angiogenic factors were measured by qRT-PCR, with values normalized to GAPDH expression. *** *p* < 0.001, ** *p* < 0.01; ns, no significance; *n* = 3 per group. Abbreviations: EF1α, elongation factor-1α; GFP, green fluorescent protein; HA-L, left homology arm; HA-R, right homology arm; PGK, phosphoglycerate kinase; Puro, puromycin; qRT-PCR, quantitative reverse transcription polymerase chain reaction; A/GP, ASCs overexpressing GCP-2 and PDGF-β; 3D-A, 3D-cultured ASCs; 3D-A/P, 3D-cultured ASCs overexpressing PDGF-β; 3D-A/GP, 3D-cultured ASCs overexpressing GCP-2 and PDGF-β.

To generate a cell line overexpressing GCP-2/PDGF-β by gene editing, we utilized transcription activator-like effector nucleases (TALENs). The donor plasmid consisted of a GCP-2/PDGF-β cassette controlled by a phosphoglycerate kinase (PGK) promoter and an elongated factor-1α (EF1α) promoter driving green fluorescent protein (GFP)-T2A-puromycin (Figure 1A). We transfected ASCs with TALENs and the donor plasmid. Following transfection, we used puromycin selection to enrich the <10% GFP-positive transfected ASCs to 40.5% (puromycin-positive) in ASCs. Fluorescence-activated cell sorting (FACS) was performed to purify the transfected ASCs, which also revealed >99.0% GFP-positive ASCs (Figure 1B). Touchdown PCR was subsequently performed to confirm the genomic integration of the donor plasmid in ASCs overexpressing GCP-2/PDGF-β (A-GP), utilizing the correct 5′ junction product (960 bp), as indicated (Figure 1C). The qRT-PCR results showed that the expression levels of GCP-2 and PDGF-β were significantly increased in A/GP compared to the control ASC (Figure 1D), indicating successful generation of the A/GP cell line. Figure 1E shows the GFP-expressing 3D-spheroid-cultured A/GP (3D-A/GP). ASCs overexpressing GCP-2 (A/G) and ASCs overexpressing PDGF-β (A/P) cell lines were also generated using the same TALEN method.

Next, to evaluate the GCP-2 or PDGF-β expression and angiogenic potential of the 3D-A/GP, we examined the expression of proangiogenic genes in the 3D-A/GP using qRT-PCR. Overall, the expression levels of the proangiogenic genes GCP-2, PDGF-β, FGF-2, HGF, IGF-1, IL-8, and VEGF-A were significantly increased in the 3D-A/GP compared to PDGF-β-overexpressing 3D spheroid ASCs (3D-A) (Figure 1F). Additionally, the expression of these angiogenic genes was confirmed using an enzyme-linked immunosorbent assay (ELISA) (Supplementary Figure S4).

### 2.2. Factors Secreted from 3D-A/GP Stimulate Cell Migration and Endothelial Tube Formation

**Figure 2:**
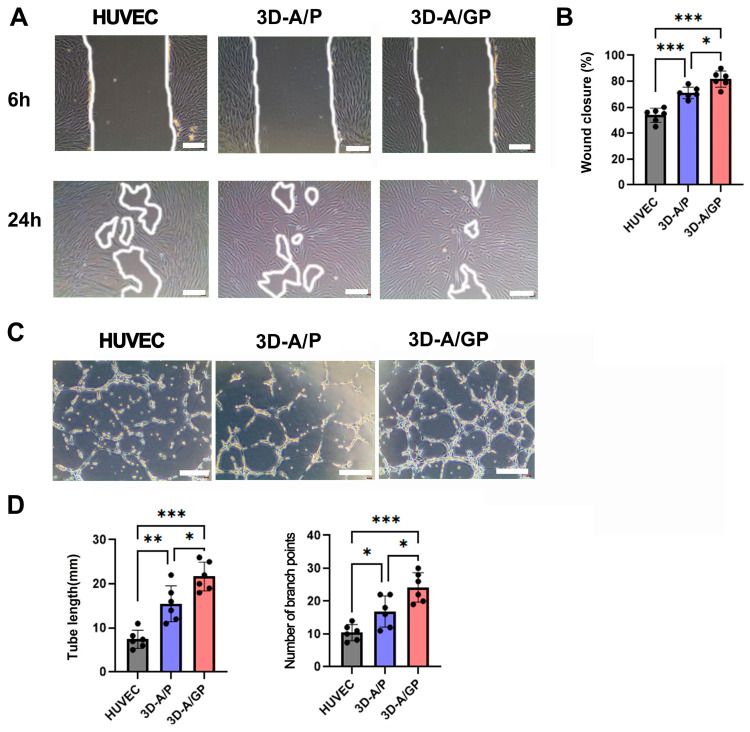
In vitro migration and angiogenic properties. (**A**) Representative photograph of scratch wound migration. Bars = 100 μm. (**B**) Culture medium (CM) of 3D-A/GP highly promoted wound closure of human dermal fibroblasts (HDFs) when compared with the CM of 3D-A/P. *** *p* < 0.001, * *p* < 0.05; *n* = 5 per group. (**C**) Representative photograph of Matrigel tube formation. Bars = 100 μm. (**D**) Quantification of branching point and tube length. CM of 3D-A/GP significantly increased the formation of tubular structure when compared with CM of 3D-A. *** *p* < 0.001, ** *p* < 0.01, * *p* < 0.05; *n* = 5 per group. Abbreviations, human vascular endothelial cells (HUVEC), PDGF-β overexpressing 3D spheroid-ASCs (3D-A/P), GCP-2/PDGF-β overexpressing 3D spheroid-ASCs (3D-A/GP).

To evaluate the ability of proteins secreted from 3D-A/GP to induce cell migration or endothelial tube formation, the scratch wound closure assay and Matrigel tube formation assay were completed. First, 3D-A/GP, 3D-A/P, and HUVECs were grown in a suspension cell culture system (low-glucose DMEM, 1% FBS) for 5 days, and culture medium (CM) was obtained from both conditions for analysis. As shown in Figure 2A,B, the scratch wound closure indicates that CM from 3D-A/GP also induced migration of fibroblasts compared to migration in CM of 3D-A/P or HUVECs. Similarly, the scratch wound closure indicates that CM from 3D-A/GP also significantly induced migration of fibroblasts when compared to their migration in CM of 3D-A/P or HUVECs (Figure 2C,D). This data indicates that factors secreted from 3D-A/GP have greatly enhanced potential to induce angiogenesis and cell migration compared to factors from 3D-A/P or HUVECs.

### 2.3. The 3D-A/GP Possess the Capacity of Vessel Formation In Vivo

**Figure 3:**
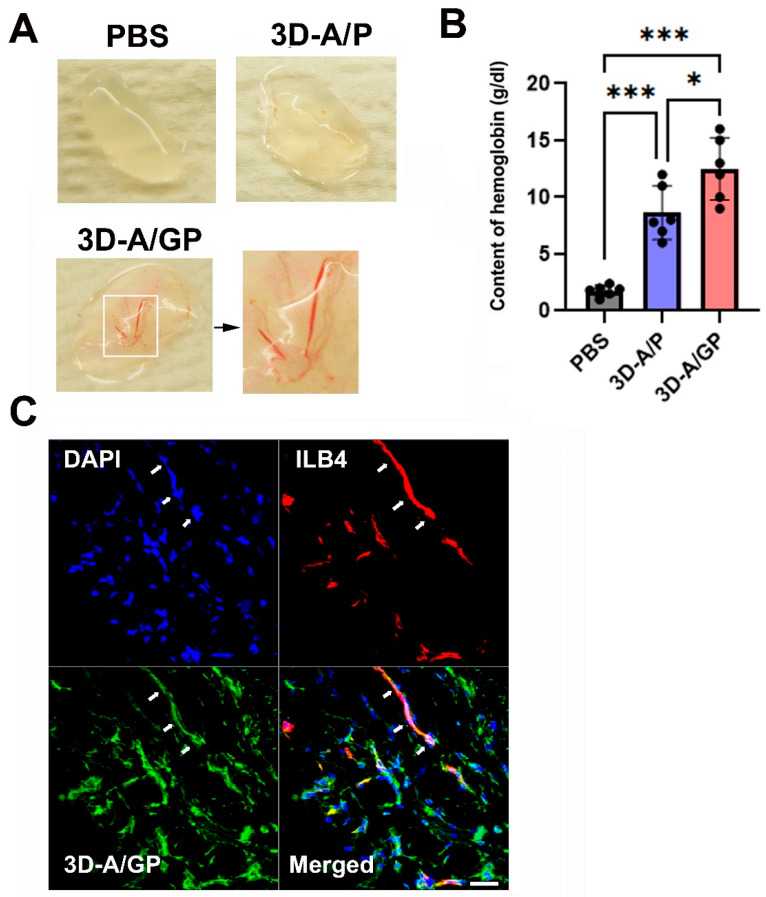
In vivo angiogenic property. (**A**) Representative photograph of Matrigel plugs injected with 3D-A/GP and 3D-A/P at 14 days after cell injection. (**B**) Quantification of hemoglobin content. The 3D-A/GP injection significantly increased the hemoglobin content when compared with 3D-A/P. *** *p* < 0.001, * *p* < 0.05; *n* = 6 per group. (**C**) Representative endothelial-cell-like differentiation of 3D-A/GP in Matrigel plugs. Bars = 50 μm. Abbreviations, Phosphate-Buffered Saline (PBS), 4′,6-diamidino-2-phenylindole (DAPI), PDGF-β overexpressing 3D spheroid-ASCs (3D-A/P), GCP-2/PDGF-β overexpressing 3D spheroid-ASCs (3D-A/GP), isolectin B4 (ILB4).

To assess the in vivo vasculogenic ability of 3D-A/GP, a Matrigel plug assay was conducted. For each of the 3D-A/GP or 3D-A/P or Phosphate-Buffered Saline (PBS) (50 μL), in the case of controls, we mixed 1 × 106 cells with Matrigel (500 μL) and subcutaneously implanted them. After 2 weeks, the Matrigel plugs were excised, and the hemoglobin content was assayed. Notably, Matrigel plugs containing 3D-A/GP had a greater number of red blood cells when compared to plugs containing 3D-A/P or PBS (Figure 3A,B), which suggests the presence of functional blood vessels. To determine whether 3D-A/GP differentiates into vasculature in the Matrigel plug, an immunohistochemistry assay was performed. The histological data were evaluated via three-dimensional confocal microscopy. The GFP-expressing 3D-A/GP developed vessel-like structures and expressed the endothelium-specific protein isolectin B4 (ILB4; red color), demonstrating differentiation of endothelial cells (Figure 3C).

### 2.4. Therapeutic Potential of 3D-A/GP in Hind Limb Ischemia (HLI) Model

**Figure 4:**
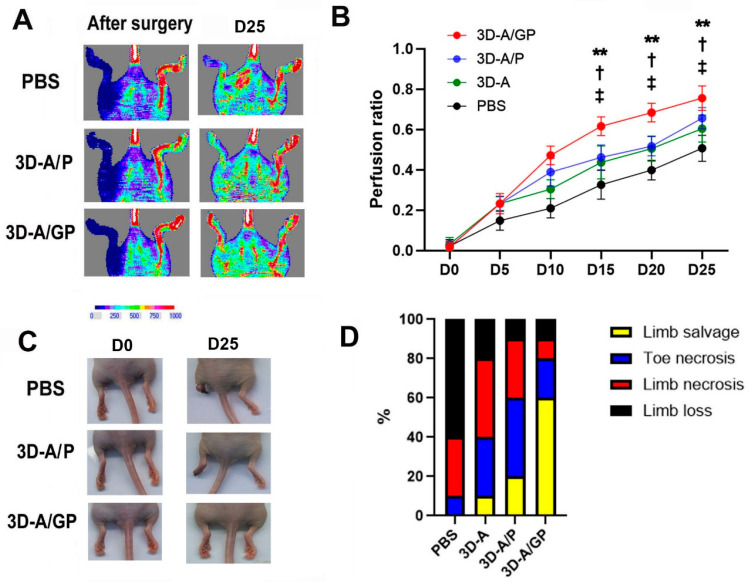
Analysis of therapeutic effects in an ischemic hind limb model. (**A**) Representative LDPI images depicting the measurement of blood flow recovery in ischemic hind limbs following cell injection. (**B**) Quantitative analysis of blood perfusion conducted 5 days after cell injection. ** *p* < 0.01, 3D-A/P vs. 3D-A/GP; † *p* < 0.05, 3D-A vs. 3D-A/GP; ‡ *p* < 0.05, PBS vs. 3D-A/GP; *n* = 6 per group. (**C**) Representative pictures of hind limbs after cell transplantation. (**D**) Quantitative analysis of limb loss, limb salvage, and limb necrosis after cell transplantation. Abbreviations, Phosphate-Buffered Saline (PBS), PDGF-β overexpressing 3D spheroid-ASCs (3D-A/P), GCP-2/PDGF-β overexpressing 3D spheroid-ASCs (3D-A/GP), 3D spheroid-ASCs (3D-A), Day 0 (D0), Day 25 (D25), Laser Doppler perfusion imager (LDPI).

To test the therapeutic value of 3D-A/GP in ischemic injury, we induced HLI surgically in nude mice. After the ligation of the left femoral artery, we injected 3D-A, 3D-A/P, or 3D-A/GP (each 1 × 10^6^ cells) or PBS intramuscularly into the ischemic hind limb. At 5 days post cell treatment, we measured the recovery rate of blood perfusion in the hind limb using laser doppler perfusion imaging (Figure 4A,B) and found that 3D-A/GP-treated limbs had a statistically significant greater recovery rate of blood perfusion compared to both the 3D-A/P and PBS limbs. Limb salvage analysis was also performed, showing there was a significant reduction in limb loss as well as a greater limb salvage ratio for the 3D-A/GP-injected mice compared to the 3D-A/P or PBS groups at 25 days after injection (Figure 4C,D).

### 2.5. Injection of 3D-A/GP Enhances Capillary Density in HLI

**Figure 5:**
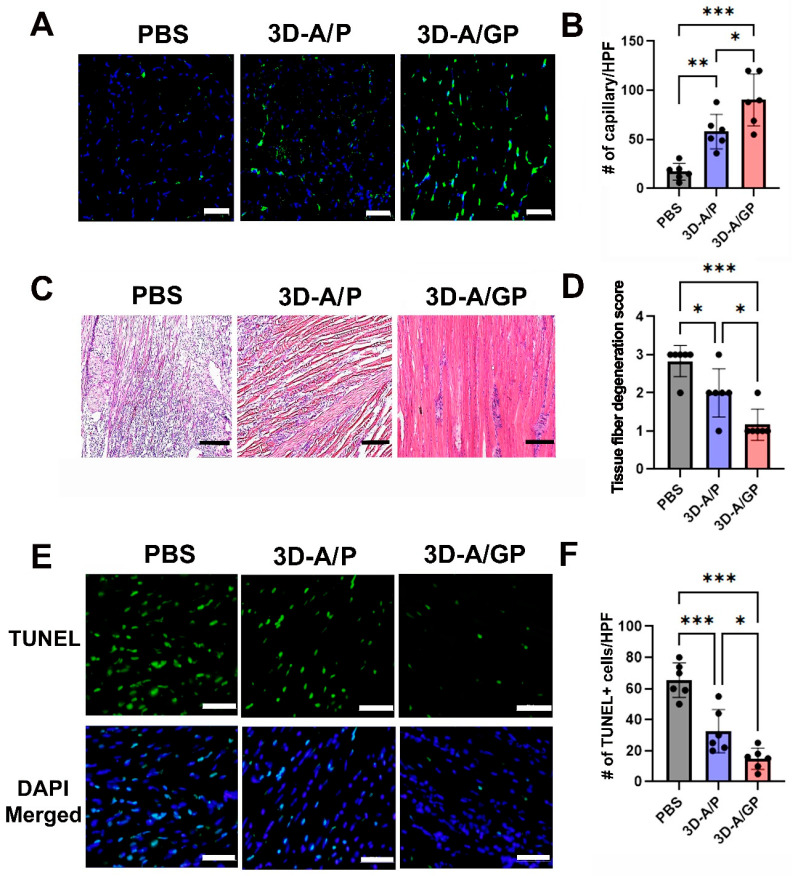
Analysis of the therapeutic mechanism. (**A**) Representative images showing the capillary density in hind limb tissues at 25 days after cell injection. The blue stain represents nuclear 4′,6-diamidino-2-phenylindole (DAPI), while the green stain represents isolectin B4 (ILB4). Bars = 500 μm. (**B**) Quantitative analysis of capillary density in hind limb tissues after cell injection. Statistical analysis shows significant differences between groups. *n* = 6 per group. *** *p* < 0.001,** *p* < 0.01 and * *p* < 0.05. (**C**) Histological images of hind limb tissues stained with hematoxylin and eosin (H&E) at 25 days after cell injection. Bars = 200 μm. (**D**) Quantitative evaluation of histological parameters in ischemic muscle tissues. *n* = 6 per group. *** *p* < 0.001 and * *p* < 0.05. (**E**) Representative images of TUNEL-positive (green) cells in the hind limb tissue after cell injection. (**F**) Quantitative analysis of TUNEL-positive (green) cells in hind limb tissues after cell injection. *** *p* < 0.001, * *p* < 0.05; *n* = 6 per group. Abbreviations, Phosphate-Buffered Saline (PBS), terminal deoxynucleotidyl transferase-mediated dUTP nick end-labeling (TUNEL), PDGF-β overexpressing 3D spheroid-ASCs (3D-A/P), GCP-2/PDGF-β overexpressing 3D spheroid-ASCs (3D-A/GP), 3D spheroid-ASCs (3D-A), High power field (HPF).

To elucidate the mechanism responsible for the improved blood flow recovery, lower rate of lower limb loss, and higher rate of limb salvage following transplantation of 3D-A/GP, capillary density was measured by histological staining with an endothelial cell marker, ILB4. Capillary density was greater in 3D-A/GP-injected mice than in 3D-A/P-injected mice and PBS-treated control mice at 25 days post injection (Figure 5A,B). The results of the hemolysin and eosin (H&E) staining demonstrated that the 3D-A/GP transplant led to significantly lower levels of tissue fiber degeneration, including reduced inflammatory cell infiltration, compared to the PBS-treated control and 3D-A/P-treated mice (Figure 5C,D). Next, to examine the anti-apoptotic potential, a TUNEL assay was performed on tissue sections taken on day 28 from the ischemic hindlimbs (Figure 5E,F). The number of TUNEL-positive nuclei in the hindlimbs was significantly less in the 3D-A/GP-treated group compared to in the PBS-treated control or the 3D-A/P-treated group.

### 2.6. The 3D-ASCs Differentiated into Endothelial-like Cells in Vivo

**Figure 6:**
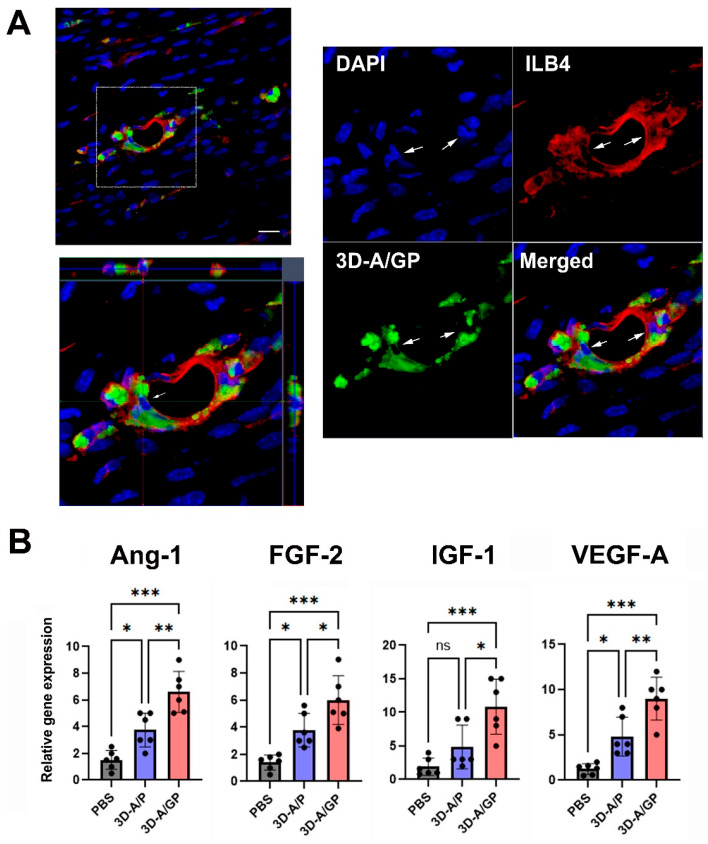
Endothelial differentiation and angiogenic property. (**A**) Three-dimensional z-stacked orthogonal representative images show co-localization at vascular-like structures with ILB4 (red) and 3D-A/GP (GFP, green) in the ischemic hind limb tissue 25 days after cell injection. Nuclei are stained with DAPI (blue). Arrows indicate the endothelial differentiation of 3D-A/GP. Scale bar = 20 μm. (**B**) Angiogenic gene expression measured by qRT-PCR. Tissues injected with 3D-A/GP exhibited elevated expression of angiogenic factors. *** *p* < 0.001, ** *p* < 0.01, * *p* < 0.05, no significance (ns); *n* = 6 per group. Next, to further evaluate the therapeutic mechanisms resulting in the increased rate of limb salvage, we evaluated the expression of angiogenic factors in hind limb tissues after cell injection using qRT-PCR. The findings indicated that the angiogenic factors angiopoietin-1 (Ang-1), fibroblast growth factor-2 (FGF-2), insulin-like growth factor-1 (IGF-1), and vascular endothelial growth factor-A (VEGF-A) were significantly upregulated in the limbs injected with 3D-A/GP compared to the 3D-A/P- or PBS-treated hind limb tissues measured 5 d after cell injection. Collectively, these data suggest that injections of 3D-A/GP stimulated an array of angiogenic biological factors for vascular protection or regeneration in the injury location of the hind limb (Figure 6B). Abbreviations, Phosphate-Buffered Saline (PBS), PDGF-β overexpressing 3D spheroid-ASCs (3D-A/P), GCP-2/PDGF-β overexpressing 3D spheroid-ASCs (3D-A/GP), 4′,6-diamidino-2-phenylindole (DAPI), isolectin B4 (ILB4), Angiopoietin-1 (Ang-1), Fibroblast Growth Factor-2 (FGF-2), Insulin-like Growth Factor-1 (IGF-1), Vascular Endothelial Growth Factor-A (VEGF-A).

To evaluate the endothelial differentiation of 3D-A/GP, we conducted an in vivo study in a mouse model of induced HLI. In this case, GFP-expressing 3D-A/GP were injected into the adductor muscle of the ischemic hind limb. After 25 days, tissues were harvested from the hind limb muscle to evaluate differentiation. Immunohistochemical analysis revealed that the injected 3D-A/GP co-localized with ILB4, an endothelium-specific marker, within the vascular-like structures of hindlimb tissue (Figure 6A). Overall, these data suggest that the injected 3D-A/GP differentiated into endothelial cells in vivo.

## 3. Discussion

The co-overexpression of GCP-2 (CXCL6) and PDGF-β in 3D ASC spheroids offers a mechanistic basis for their sequential synergistic amplification of proangiogenic signaling. Indeed, GCP-2 signaling through CXCR1/2 has been shown to activate critical ERK1/2, NF-κB [12], which is known to upregulate VEGF-A, MMP-2/9, and SDF-1, facilitating endothelial migration, proliferation, and extracellular matrix remodeling [5]. More importantly, we reported previously that GCP-2 overexpression increases the expression of a variety of angiogenic factors, such as VEGF-A, HGF, IL-8, and IGF-1 [5]. Concurrently, PDGF-β activates PDGFR-β and activates the PI3K/Akt and MAPK/ERK pathways that increase the proliferation and motility of mesenchymal stromal cells, as well as recruit pericytes in addition to stabilizing newly formed vessels [13]. Based on this, we speculate that the beneficial effects of GCP-2 with PDGF-β will be higher than the co-administration of PDGF-β and VEGF, which has been previously reported to have a greater impact on increased density and maturity of angiogenic vessels than either factor used alone [14]. Furthermore, 3D culture of ASCs has been found to support the release of angiogenic and anti-inflammatory factors, such as FGF-2, EGF, and IL-10 [11]. Three-dimensional culture is more relevant to in vivo conditions as it allows for hypoxia-induced stabilization of HIF-1α, which is essential for the expression of proangiogenic genes and the paracrine release of angiogenic and protective factors [15]. Therefore, our hypothesis is that both GCP-2 and PDGF-β, when simultaneously overexpressed within 3D spheroids, can be used as a novel, effective, safe, and strong proangiogenic therapeutic.

Conditioned medium from 3D-A/GP showed superior functionality over other single-factor controls, as evidenced by enhanced fibroblast migration and endothelial tube formation in vitro. These results are consistent with studies that indicate that simultaneous delivery of multiple angiogenic factors resulted in larger and more mature neovascular networks than when each cytokine was delivered separately [16]. The architecture of the 3D spheroids may add to this effect by enabling greater paracrine secretion. This is the first evidence to demonstrate that GCP-2/PDGF-β co-expression in 3D ASC spheroids has therapeutic potential in regenerative vascular applications.

In vivo results supplied via Matrigel plug experiments corroborated that 3D-A/GP spheroids not only secrete strong angiogenic factors but also participate in new vessel formation. Plugs containing 3D-A/GP had significantly higher hemoglobin content and more red blood cell invasion than plugs containing either 3D-A/P or PBS, providing evidence of functional microvessels forming. Furthermore, the results of confocal immunohistochemistry showed that the 3D-A/GP, labelled with GFP, organized into lumen-like structures and positively expressed ILB4, considered the endothelial-specific marker, indicating direct differentiation into vascular endothelium. These results corroborate previous reports demonstrating the ability of stem cell-based scaffolds to promote both paracrine-driven neovascularization and direct endothelial incorporation [17].

Our work in vivo with the HLI model demonstrated that the recovery and limb salvage from hind limb ischemia increased significantly when the 3D-A/GP spheroids were administered intramuscularly. Laser Doppler imaging performed on day 5 post treatment showed significantly greater blood flow in the 3D-A/GP-injected limbs than in the 3D-A/P or PBS control conditions, and by week 4, these animals had a substantially lower incidence of necrosis and amputation. The increased efficacy of our dual GCP-2/PDGF-β spheroids was likely due to the combined chemotactic and vessel-stabilizing cues provided, a notion that has induced more durable neovascular networks in preclinical HLI models than single-factor approaches [18]. Overall, these results highlight the potential for 3D-A/GP constructs to be translated into an effective cell-based therapy for peripheral arterial disease. Moreover, the ability of the spheroids to elicit both functional blood flow recovery and tissue salvage in the setting of severe ischemia further emphasizes their potential for clinical translation.

Our histological findings revealed that the capillary density in ischemic muscles was significantly greater in mice treated with 3D-A/GP. This increase coincided with a marked reduction in myofiber degeneration and inflammatory cell infiltration, as observed in H&E-stained sections. Furthermore, a significant decrease in the TUNEL-positive nuclei indicates powerful anti-apoptotic effects. Mechanistically, 3D-A/GP co-expressed endothelial cell markers within vessel-like structures, supporting the conclusion that mesenchymal stem cells differentiated into endothelial cells in vivo. Furthermore, quantitative RT-PCR analysis demonstrated a significant upregulation of Ang-1, FGF-2, IGF-1, and VEGF-A, which are well-recognized for their roles in vessel maturation, endothelial cell proliferation, matrix remodeling, cytoprotection against ischemic apoptosis, and capillary sprouting. These findings are consistent with a previous report, which showed that ASCs can contribute to neovascularization and transdifferentiate into endothelial cells under appropriate angiogenic stimuli [19]. Notably, the combination of cell-autonomous differentiation and a potent paracrine secretome parallels previously related work using HLI models [20], highlighting for the first time the translational potential of using 3D-A/GP spheroids for therapeutic neovascularization in the treatment of peripheral arterial disease.

While 3D-A/GP spheroids show promise of therapeutic potential for peripheral arterial disease (PAD), there are several limitations to address to explore the full clinical potential. One of the more important issues is the potential for high levels of angiogenesis due to GCP-2 and PDGF-β being used together. Excessive angiogenic factors could cause abnormal or excessive vascular structures, as well as unlimited tissue overgrowth. Furthermore, sustained or continual overexpression of these factors may provoke inflammatory reactions or immune-mediated responses, especially in a clinical setting. Regarding PAD or diabetic wounds, future studies are needed to address the long-term stability of this approach and side effects. It is critical to demonstrate the safety and efficacy of this therapy. In addition to ischemic disorders, such as PAD or diabetic ulcers, the potential to treat myocardial ischemia may exist, in addition to utility for wound healing in the presence of robust angiogenic and regenerative factors that facilitate functional recovery. In conclusion, 3D-A/GP spheroids represent a novel development with significant translational potential for new regenerative vascular therapies to resolve a multitude of ischemic and tissue repair issues.

This study has several limitations that should be acknowledged. First, the activity of signaling pathways, including ERK1/2, NF-κB, and PI3K/Akt, was not directly assessed. As a result, the mechanistic explanation for the observed phenomena should be interpreted cautiously in the absence of definitive validation. Second, the study lacks comparative data to determine whether the elevated levels of GCP-2 and PDGF-β remain within the physiological range of upregulation typically observed in native human or murine tissues. Without such data, the translational insights are limited, and concerns about potential off-target effects, such as aberrant neovascularization, cannot be comprehensively addressed. Third, although GFP-positive transplanted cells were found to co-localize with ILB4, we did not quantify how many of these cells acquired endothelial-like characteristics, nor did we perform functional assays to confirm their endothelial identity. Finally, only young male nude mice were used in the HLI model; considering known sex differences in angiogenesis and ischemic recovery, further studies using female mice will be necessary to strengthen the findings.

## 4. Materials and Methods

### 4.1. Cell Culture

Human adipose-derived stem cells (ASCs), human vascular endothelial cells (HUVECs), and human dermal fibroblasts (HDFs) were obtained from Thermo Scientific Inc. (Rockford, IL, USA). To form 3D spheroids, cells (5 × 10^4^ cells) were suspended in 25 µL of culture medium containing 10% fetal bovine serum (FBS), α-MEM, 100 U/mL penicillin, and 100 mg/mL streptomycin, as previously reported [21]. The cell suspension was transferred to the lids of culture plates and maintained in an inverted position for five days to allow spheroid formation. Spheroids were dissociated by incubating them with 0.05% trypsin/EDTA for 5 min, after which the enzymatic activity was halted with fresh culture medium. These dissociated cells were then used for subsequent experiments.

### 4.2. Donor Vector Construction

The genes for GCP-2/PDGF-β, GCP-2, or PDGF-βwere synthesized and inserted into the adeno-associated virus integration site 1 (AAVS1) safe harbor site of the targeting donor vector (System Biosciences, Palo Alto, CA, USA), using Nde I and Sal I restriction sites (Supplementary Figure S1) [22].

### 4.3. Transfection and Selection and Fluorescence-Activated Cell Sorting (FACS)

ASCs were cultured in DMEM supplemented with 10% FBS. For electroporation, ASCs were harvested, counted, and resuspended at a concentration of 1 × 10^5^ cells in 10 μL of electroporation buffer with 0.6 μg of each of the following: AAVS1 left TALE-Nuclease vector, AAVS1 right TALE-Nuclease vector, and AAVS1 HR Donor (System Biosciences, Palo Alto, CA, USA) (Supplementary Figure S1). Electroporation was conducted using the Neon Transfection System (Thermo Fisher, Waltham, MA, USA). Five days post transfection, GCP-2/PDGF-β, GCP-2, or PDGF-β knock-in cells were selected by incubating them with 5 μg/mL puromycin for seven days. Puromycin-resistant cells were subsequently resuspended in FACS buffer and sorted according to standard protocols (Supplementary Figure S2) [23].

### 4.4. Genomic DNA Extraction and Junction PCR

Genomic DNA was extracted from cultured cells using the G-spin™ Total DNA Extraction Mini Kit (Intron Biotechnology, Seongnam, Republic of Korea), following the manufacturer’s protocol. A total of 120 ng of genomic DNA was then subjected to touchdown PCR for 36 cycles, followed by a secondary PCR, as previously outlined [23]. The amplified genomic DNA band was visualized using ethidium bromide and photographed with a Chemi Doc XRS imaging system (Bio-Rad Laboratories, Hercules, CA, USA) (Supplementary Figure S3).

### 4.5. Conditioned Medium (CM) Collection

To collect conditioned media (CMs), the 3D spheroid cells were cultured in glucose-containing DMEM with 10% FBS, 100 U/mL penicillin, and 100 mg/mL streptomycin (Gibco, Thermo Fisher, Waltham, MA, USA), using a spinner flask (Thermo Fisher, Waltham, MA, USA) and magnetic stirring for three days. The culture media were centrifuged at 1000× *g* for 10 min to remove cell debris, and the supernatants were collected for further experiments. For cell dissociation, spheroids were treated with 0.05% trypsin/ethylenediaminetetraacetic acid for five minutes, after which trypsinization was halted by adding fresh α-MEM with 10% FBS, 100 U/mL penicillin, and 100 mg/mL streptomycin.

### 4.6. Gene Analysis

The quantitative real-time polymerase chain reaction (qRT-PCR) was performed as previously described [24]. Total RNA was extracted from each cell sample using RNA-stat (Iso-Tex Diagnostics, Friendswood, TX, USA), and reverse transcription was carried out using TaqMan Reverse Transcription Reagents (Applied Biosystems, Foster City, CA, USA) following the manufacturer’s guidelines. The synthesized complementary DNA (cDNA) was amplified using qRT-PCR with human- or mouse-specific primers and probes (Table 1) on an ABI PRISM 7000 Sequence Detection System (Applied Biosystems, ThermoFisher, Waltham, MA, USA). Relative mRNA expression levels were normalized to GAPDH as a reference gene. All primers and probes were purchased from Applied Biosystems.

### 4.7. Matrigel Tube Formation Assay

To evaluate tube formation capability, conditioned media (CMs) were prepared using modified previous methods. HUVECs at a density of 1 × 10^4^ cells per well were cultured in low-glucose DMEM (Gibco, ThermoFisher, Waltham, MA, USA) containing 1% FBS (control) or CM from the stromal vascular fraction (SVF) and ASCs on Matrigel-coated glass slides (NUNC, ThermoFisher, Waltham, MA, USA). After a 5-h incubation, representative fields were randomly imaged under an inverted microscope, and the tube length and branching points were quantified as previously described [24].

### 4.8. Scratch Migration Assay

The scratch migration assay was conducted following established methods [25]. Briefly, HDFs were seeded at 1 × 10^5^ cells per well in 24-well plates and incubated at 37 °C with 5% CO_2_ for 27 h to form confluent monolayers. A scratch was created in each monolayer using a sterile pipette tip, and cells were treated with CM from each cell type. Images of wound closure were captured at five random locations, and wound area analysis was performed using NIH Image software (http://rsb.info.nih.gov/nih-image/, accessed on 1 February 2025).

### 4.9. Matrigel Plug Assay

To investigate the in vivo vasculogenic potential, we performed a Matrigel plug assay following the procedure outlined by Jeong et al. [25]. Specifically, 2 × 10^5^ cells were mixed with 500 μL of Matrigel and subcutaneously transplanted into nude mice. After 14 days, the Matrigel plugs were collected, and the hemoglobin levels were evaluated using Drabkin’s Reagent Kit (Sigma, St. Louis, MO, USA).

### 4.10. Cell Transplantation in the HLI Mouse Model

The experimental protocols used in this study were approved by the Catholic Kwandong University Institutional Animal Care and Use Committee. HLI was induced using a previously described method [24]. Briefly, male nude mice aged between seven and ten weeks, weighing 18–22 g, were used for the experiments (Joongang Laboratory Animal Inc., Seoul, Republic of Korea). Each mouse was anesthetized with isoflurane (induction: 450 mL air, 4.5% isoflurane; maintenance: 200 mL air, 2.0% isoflurane; Baxter International, Inc., Deerfield, IL, USA), and the right femoral artery was surgically ligated. Subsequently, a solution containing 1 × 10^6^ cells was intramuscularly injected into the ischemic hind limb area after surgery (*n* = 7 for each group). Euthanasia was carried out by intravenous injection of thiopental-sodium (40 mg/kg). To assess the blood flow in the hind limb over time, laser Doppler perfusion imaging (Moor Instruments, Axminster, UK) was used.

### 4.11. Histological Analysis

For histological analysis, the adductor muscles were harvested and fixed in paraformaldehyde for 4 h, followed by overnight incubation in a 15% sucrose solution. The tissues were then embedded in OCT compound (Sakura Finetek USA, Torrance, CA, USA) and sectioned into 10 μm thick sections [24]. Frozen sections of ischemic tissues were stained with biotinylated isolectin B4 (ILB4, 1:250; Vector Laboratory Inc., Burlingame, CA, USA) primary antibodies. This was followed by incubation with streptavidin Alexa Fluor 555 or 488 (1:400) (Invitrogen, Carlsbad, CA, USA) secondary antibodies for capillary density measurement. Subsequently, five fields from five tissue sections were randomly selected, and the number of capillaries was counted. In addition, sectioned samples were stained with Harris hematoxylin solution (Sigma, St. Louis, MO, USA) for 3 min followed by eosin Y (Sigma) for 25 s. The TdT-mediated dUTP nick-end labeling (TUNEL, ThermoFisher, Waltham, MA, USA) assay was also performed using a fluorescein in situ cell death detection kit (Roche Molecular Biochemical, Indianapolis, IN, USA).

### 4.12. Scoring Criteria for Histological Evaluation

The degree of muscle fiber degeneration and inflammatory cell infiltration in H&E-stained sections was measured using a 4-point scoring system. A score of 0 was assigned to sections with normal tissue architecture with no muscle fiber degeneration or inflammatory cell infiltration. A score of 1 was assigned based on mild fiber degeneration or a minimal number of infiltrating inflammatory cells. A score of 2 was noted for sections showing moderate muscle fiber degeneration or moderate infiltration of inflammatory cells. A score of 3 was given based on severe muscle fiber degeneration or severe infiltration of inflammatory cells.

### 4.13. Statistical Analysis

Data are expressed as the mean ± standard deviation. Statistical comparisons between two groups were made using the Student’s *t*-test, while multiple comparisons were analyzed using one-way analysis of variance (ANOVA) followed by Bonferroni’s post hoc test, using Graphpad version 10.0. A *p*-value < 0.05 was considered statistically significant.

## Figures and Tables

**Table 1:** Primers for qRT-PCR.

Gene	Human	Mouse
Ang-1		Mm00456503_m1
FGF-2	Hs00266645_m1	Mm00433287_m1
GAPDH	Hs99999905_m1	Mm99999915_g1
GCP-2	Hs00237017_m1	
HGF	Hs00300159_m1	
IGF-1	Hs01547657-m1	Mm00439560_m1
PDGF-β	Hs00966522_m1	
IL-8	Hs00174103_m1	
VEGF-A	Hs99999070_m1	Mm01204733_m1

## Data Availability

The original contributions presented in this study are included in the article/Supplementary Material. Further inquiries can be directed to the corresponding author.

## Supplementary Materials

The following supporting information can be downloaded at: https://www.mdpi.com/article/10.3390/ijms26178425/s1.
