# Supplementary material for: Robust Angio-Vasculogenic Properties of 3D-Cultured Dual GCP-2/PDGF-β Gene-Edited Human ASCs
Source: Int J Mol Sci. 2025 Aug 29;26(17):8425. doi: 10.3390/ijms26178425 (PMC12428705; doi:10.3390/ijms26178425)

Supplementary Data

Supplementary materials and methods

Enzyme-linked Immunosorbent Assay (ELISA)

In 10-cm culture dishes, cells (1×10^6 cells) were plated and cultured in complete medium at 37°C, under a humidified environment with 5% CO_2. After 24 hours in culture, the culture medium was replaced with serum-free medium, and the cells were incubated for a further 24 hours. The conditioned medium was harvested and centrifuged at 2,000 g for 10 minutes to remove cellular debris. The supernatant was filtered through a 0.22 μm filter unit (Millipore, Bedford, MA, USA). Concentrations of GCP-, PDGF-, HGF, IGF-1, IL-8 and VEGF-A from the conditioned medium were measured utilizing commercially available ELISA kits (Thermo Fisher Scientific Inc., Rockford, IL, USA) according to the instructions provided by the manufacturer. Absorbance was then measured at specified wavelength using a microplate reader (BioTek, USA).

Supplementary Figure Legend

Supplementary Figure S1. TALEN vector information (System Biosciences). (A) AAVS1 target sequence. (B) AAVS1 TALEN pair vector (C) AAVS1 donor vector.

Supplementary Figure S2. FACS sorting results. (A) Gating strategy to isolate GFP+ GP cells. (B) GFP+ GP cells after sorting.

Supplementary Figure S3. Donor plasmid insertion was confirmed via junction PCR. The original gel band was visualized and photographed using Chemi Doc XRS.

Supplementary Figure S4. GCP-2, PDGF- β and multiple angiogenic factors were measured by ELISA, with values normalized to GAPDH expression. *** $p < 0.001$, ** $p < 0.01$, * $p < 0.05$, ns, no significance; n = 3 per group.

Supplementary Figure S1.

A

```
AAVS1 target      - CCCCTCCACCCCACAGTGGGGCCACTAGGGACAGGATTGGTGACAGAAA -
pZT-AAVS1-TALE-N  - CCCCTCCACCCCACAGTggggccactagggacAGGATTGGTGACAGAAA -
```

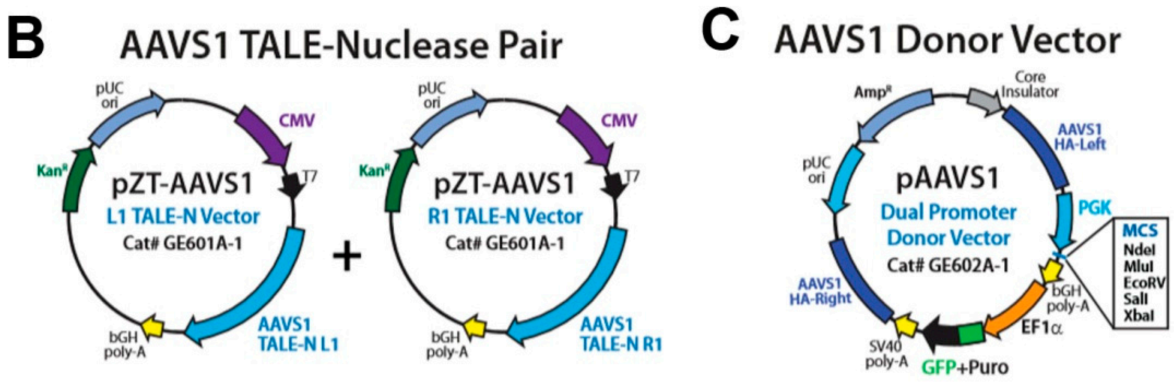

Supplementary Figure S2.

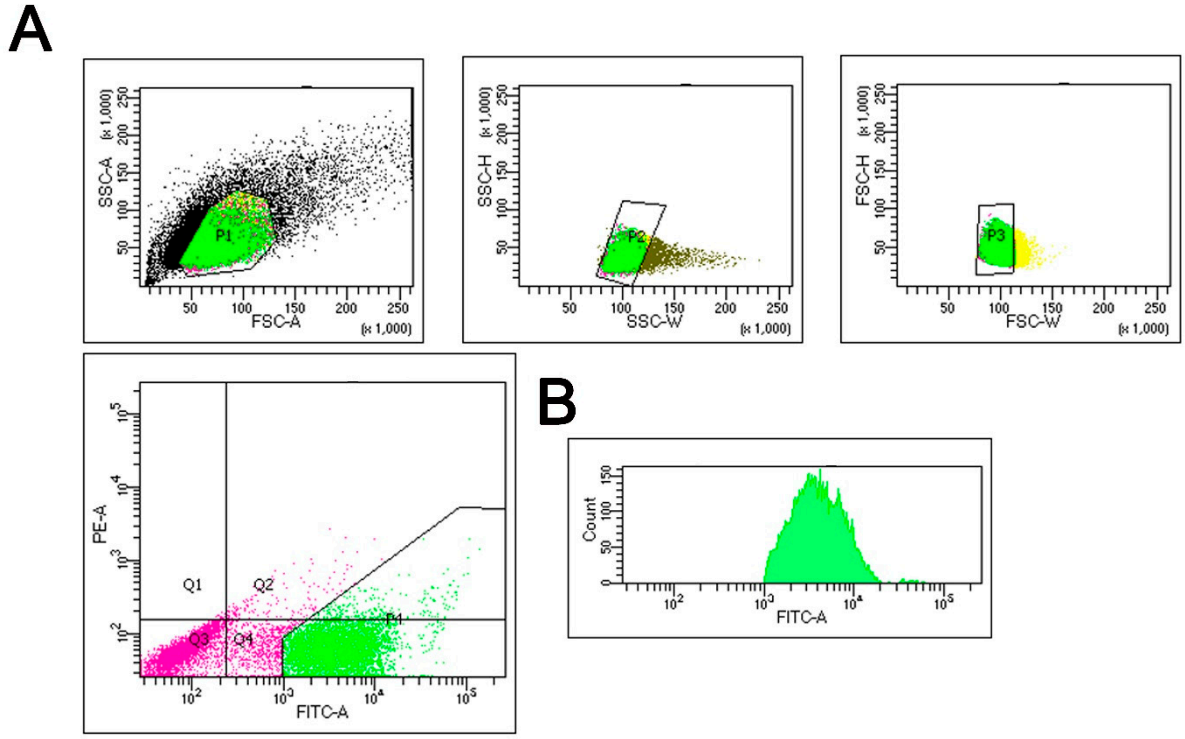

Supplementary Figure S3.

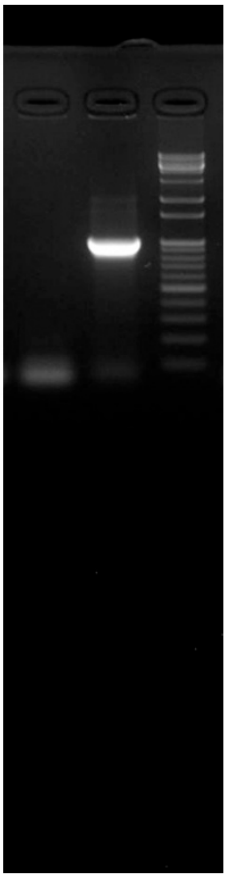

Supplementary Figure S4.

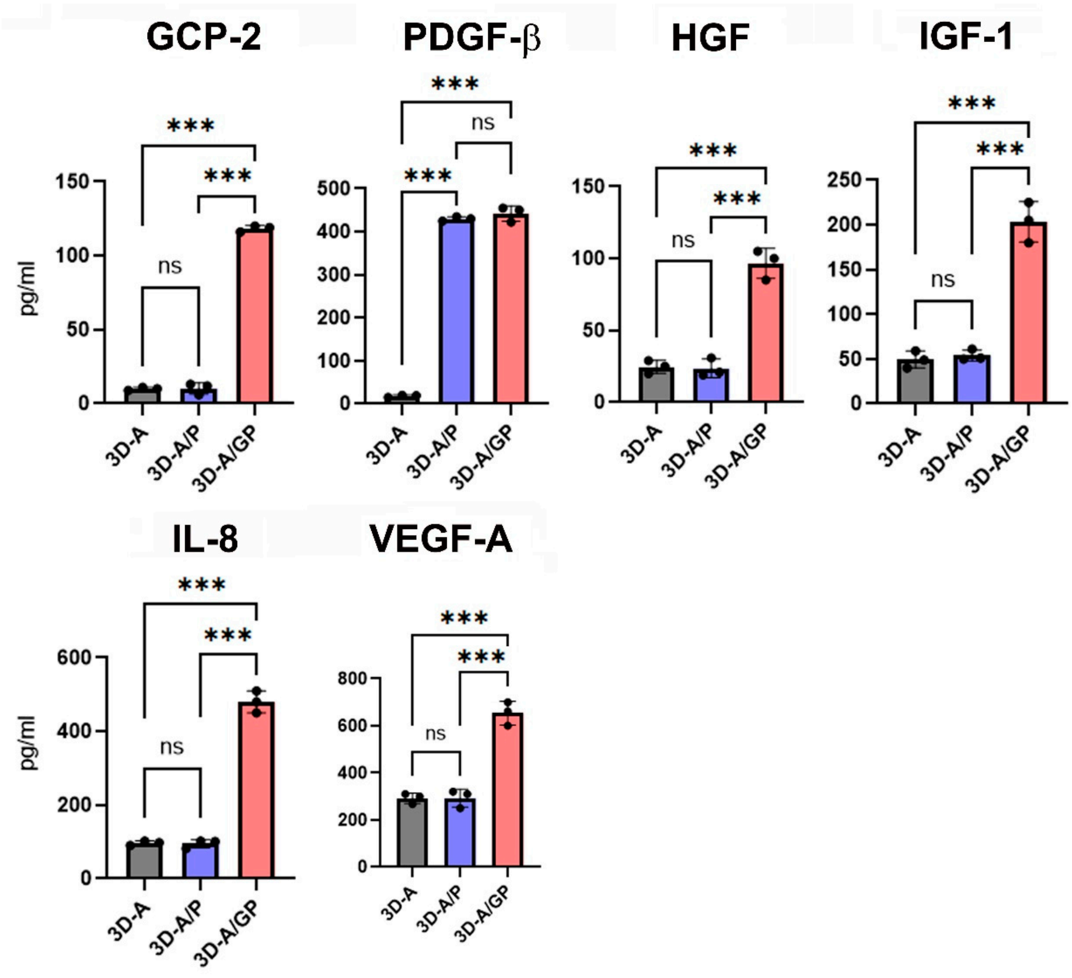

Supplement: Supplementary file 1 [file ijms-26-08425-s001.zip › ijms-3764390-supplementary.pdf]
